# Nurses' Pension Funds

**Published:** 1887-02-26

**Authors:** R. Gofton Salmond

**Affiliations:** Hon. Secretary. 73, Cheapside, E.C.


### NURSES' PENSION FUNDS.

It would appear from the report of the meeting of the Hospitals Association, at which I was unable to be present, that there is no fund in existence for giving pensions to worn-out trained nurses.

This fund (the Trained Nurses' Annuity Fund) was founded in 1874, and has been steadily increasing ever since. At present twelve annuities of £15 per annum have been permanently founded, and about £4,000 is invested in the hands of trustees, the interest of which pays the annuitants, so that there is no risk of an annuity failing through lack of funds, but the number of applicants is daily increasing. It is calculated that there are now 10,000 nurses in the United Kingdom. If each would give 1*d*. a month or 1*s*. a year towards the fund, its powers of usefulness would rapidly increase. Lady superintendents and matrons of hospitals and infirmaries are therefore earnestly requested to bring this matter before their staff, and to plead on behalf of their sisters whose health has broken down under the fatigue and anxiety of their arduous profession.

Any communications from those interested in the work can be addressed to Lady Bloomfield, Shrivenham, Berks; or to

R. GOFTON SALMOND, Hon. Secretary.

73, Cheapside, E.C.

